# A Case Report of a Spontaneous Angular Pregnancy: A Diagnostic Challenge

**DOI:** 10.7759/cureus.114940

**Published:** 2026-08-21

**Authors:** Reagan Boyett, Maria Evola Pate, Alexandra G Smith, Jessica Rodriguez, Jonathan Payne

**Affiliations:** 1 Medicine, Edward Via College of Osteopathic Medicine, Auburn, USA; 2 Medicine, Alabama College of Osteopathic Medicine, Dothan, USA; 3 Obstetrics and Gynecology, Grandview Medical Center, Birmingham, USA; 4 Obstetrics and Gynecology, Southeast Health Medical Center, Dothan, USA

**Keywords:** angular pregnancy, difficult diagnosis, early pregnancy loss, obst gynec, ultrasound

## Abstract

Angular pregnancies are rare, high-risk pregnancy complications in which the embryo implants in the angle of the uterus. The diagnosis is made by ultrasound to be distinguished from a myometrial or cornual pregnancy. We present a 31-year-old female who presented to the clinic for confirmation of pregnancy and spotting in the days prior to her appointment. The patient was followed closely in the week following her diagnosis until the pregnancy aborted. We discuss various risk factors, management options, and outcomes of patients with angular pregnancies. This case adds to the paucity of literature relating to angular pregnancies.

## Introduction

An angular pregnancy is a gestation that implants within a lateral angle of the endometrial lining of the uterus and just medial to the cornu [1]. These pregnancies are rare and high risk for both the mother and the fetus [2]. Due to the rarity of these cases, lack of physician awareness, and anatomic proximity making them difficult to distinguish from interstitial or cornual pregnancies, the true prevalence of angular pregnancies is unknown. Some sources report fewer than 100 cases in the literature [3].

Live angular pregnancies are diagnosed via ultrasound by anatomic location within the angle of the uterus, but angular pregnancy that results in spontaneous abortion can be diagnosed using hysteroscopy [4]. They most often result in a live birth, but these pregnancies can end in early pregnancy loss and uterine rupture [5]. Uterine rupture is a well-documented risk associated with angular pregnancy, though reported rates vary considerably across studies.

We report a rare case of angular pregnancy resulting in spontaneous abortion with the aim of increasing obstetrician, radiologist, and sonographer awareness of this diagnosis and encouraging clinicians to consider it in the differential when evaluating early pregnancy location.

## Case presentation

**Figure 1:**
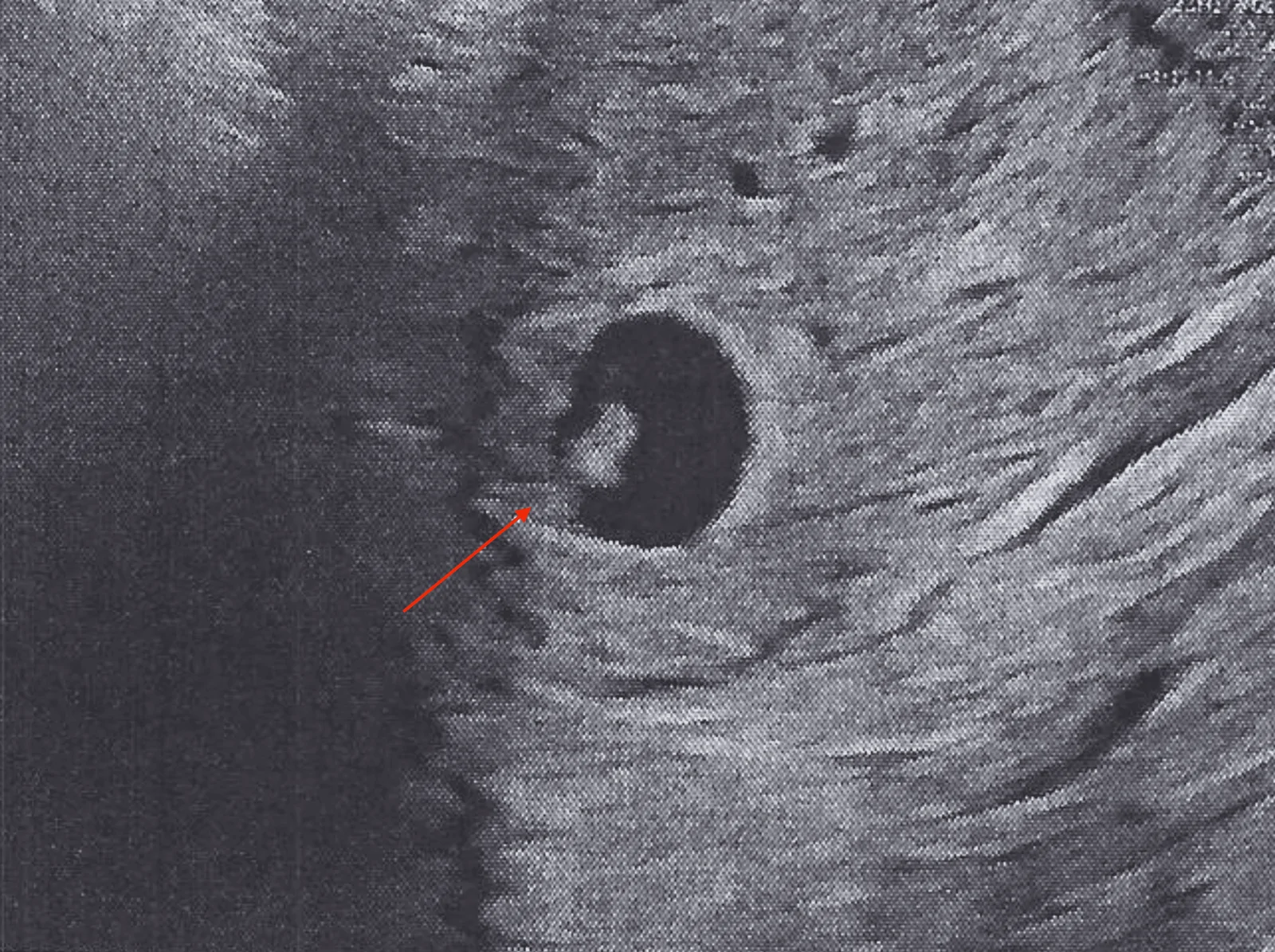
Obstetric ultrasound Obstetric ultrasound with a red arrow demonstrating a gestational age of five weeks and six days.

**Figure 2:**
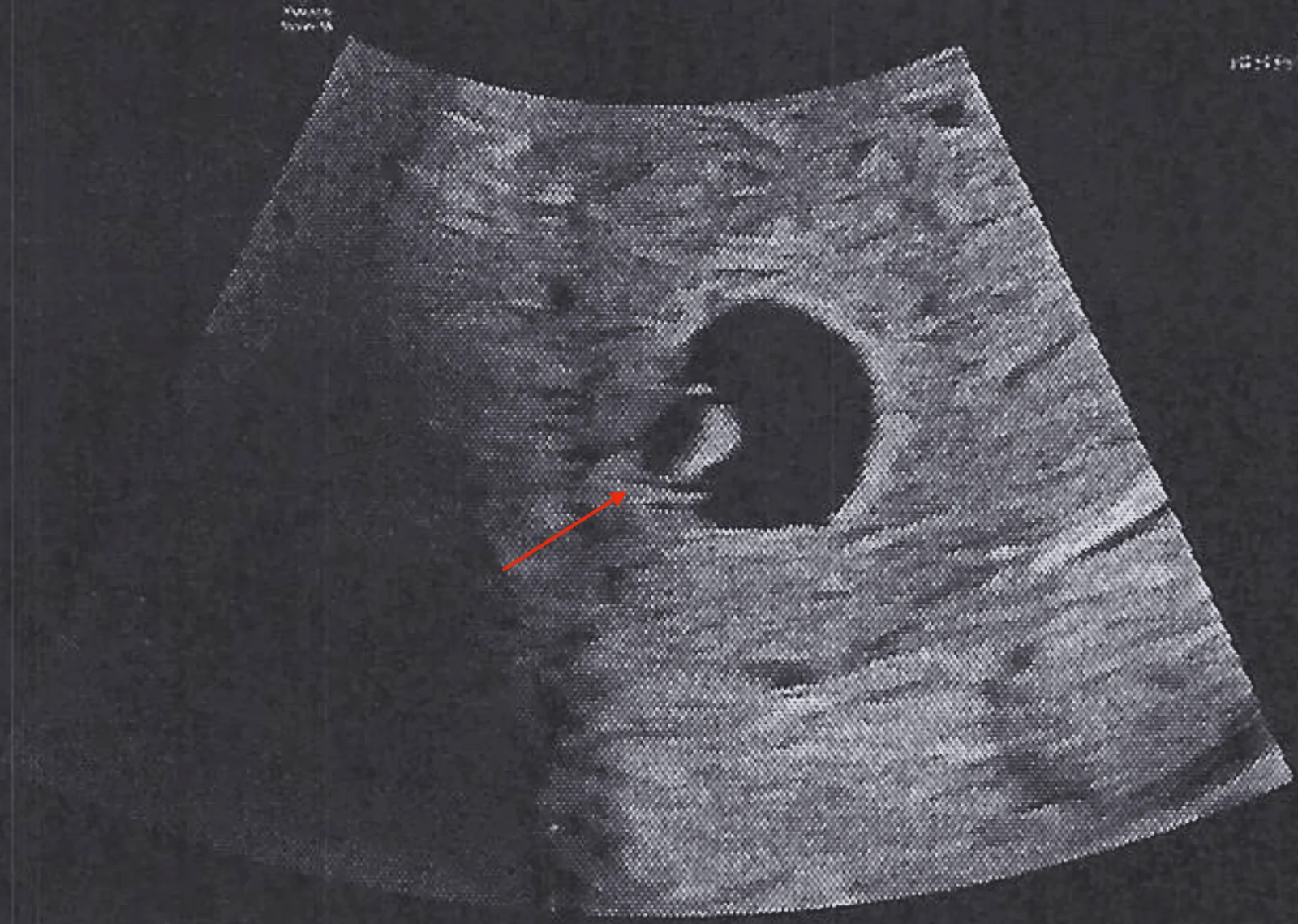
Obstetric ultrasound Obstetric ultrasound with a red arrow showing a gestation near the lateral edge of the uterus.

**Figure 3:**
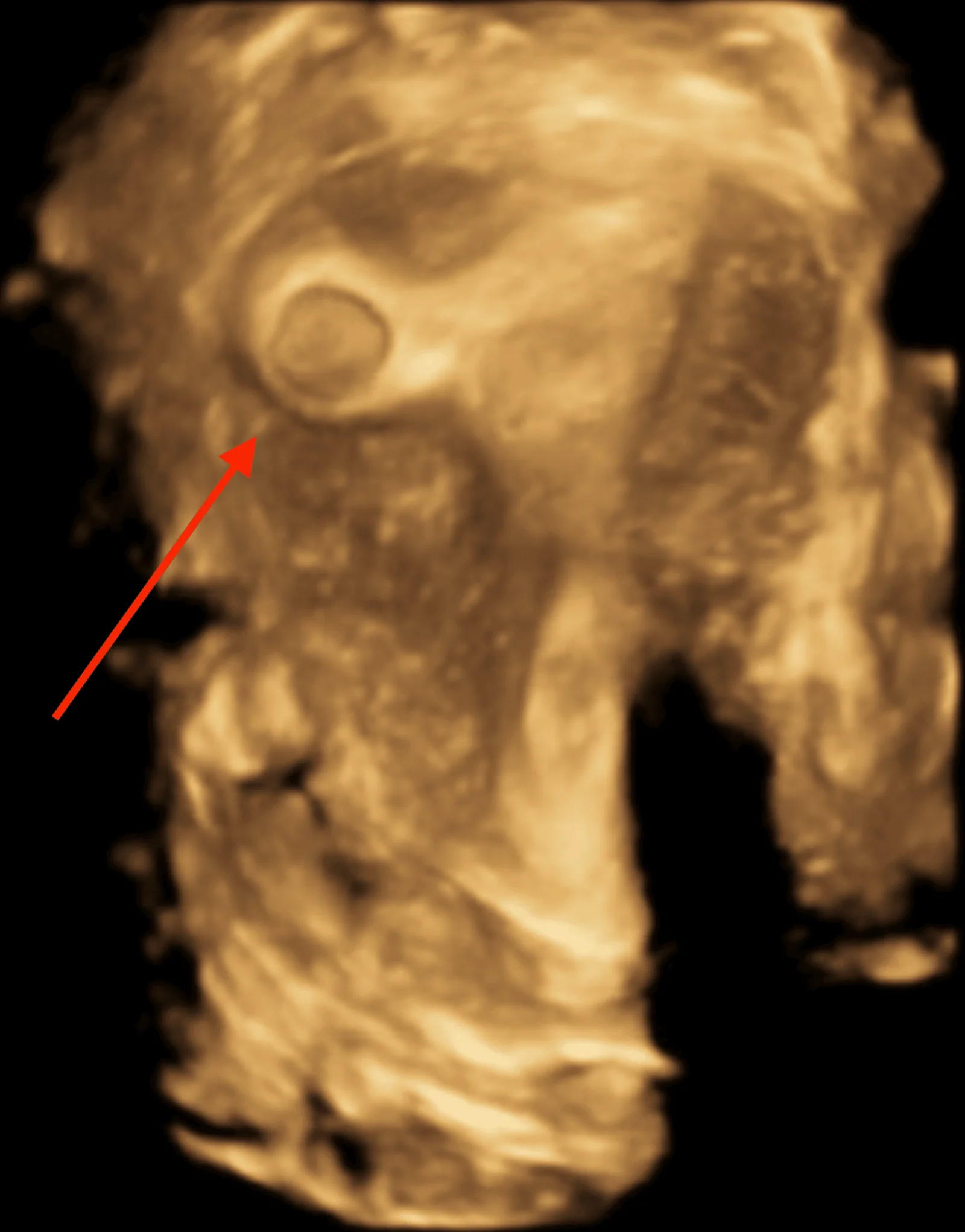
3D obstetric ultrasound 3D obstetric ultrasound with a red arrow, highlighting the location of the gestation at the angle of the uterus.

A 31-year-old primagravida female presented to the OBGYN clinic for confirmation of pregnancy after spotting over the course of the preceding week. She did not utilize assistive reproductive technology in becoming pregnant and had no history of gynecologic surgery. She has a history of irregular periods, but by her last menstrual period, she is estimated to be eight weeks gestation. At her confirmation of pregnancy appointment, the ultrasound technician estimated the gestational age to be five weeks and six days (Figures 1-3).

The physician was called in to review the imaging. The physician consulted other obstetricians and a radiologist and suspected a myometrial or cornual pregnancy. Due to the high risk and uncertainty about the true etiology of the pregnancy, the patient was referred to a specialty resident ultrasound clinic at a major academic institution that was holding appointments that morning. Upon follow-up with the patient, it was determined that the patient had an angular pregnancy. This diagnosis was made via ultrasound based on the location of the gestation at the angle of the uterus and medial to the uterotubular junction.

The patient was counseled to continue the pregnancy under supervision from the obstetric team. In the 48 hours following her confirmation of the pregnancy appointment, the patient began to spot and pass the pregnancy. The follow-up appointment at one week from her initial encounter demonstrated complete passage of the pregnancy on ultrasound.

## Discussion

Angular pregnancies are usually diagnosed via ultrasound. Bollig et al. proposed a set of sonographic criteria for diagnosis, requiring a nonanomalous uterus (excluding unicornuate, bicornuate, or septate variants), implantation of the blastocyst in the lateral angle of the uterine cavity, just medial to the uterotubal junction, and no more than 1 cm of myometrial thickness between the gestational sac and the outer uterine border. The criteria also require completely circumferential endometrium surrounding the gestational sac, confirming an intrauterine gestation, and the absence of an echogenic line bordering the gestational sac in the upper lateral uterus [6]. This set of criteria has not been adopted or enforced by current guidelines. Commonly, these pregnancies are mistaken for cornual, interstitial, or, in our case, intramural pregnancies.

In a recent study, Chen et al. examined whether embryo- and cycle-level factors influence the risk of angular pregnancy following assisted reproductive technology (ART). Their multivariable regression analysis concluded that multiple embryo transfer may increase the incidence of angular pregnancy in ART-conceived pregnancies [7]. Other risk factors for angular pregnancy may include uterine anomalies.

Angular pregnancies that progress to term have been reported to carry an increased risk of intrauterine growth restriction, as well as complications in the third stage of labor, particularly retained placenta. Due to the lack of standardized diagnostic criteria and treatment, management of these complications remains challenging [8]. Most case reports and case series tend to favor expectant management [6,9,10]. In the event of gestational demise, some cases have described the hysteroscopic management of an angular pregnancy, illustrating that hysteroscopy can serve as both a diagnostic and therapeutic modality, allowing direct visualization and minimally invasive, intact removal of the gestational sac [4,11].

These pregnancies are diagnosed at a mean gestational age of 7.4 weeks [6]. Boling et al. followed up two weeks after diagnosis and found that 55% of previously diagnosed angular pregnancies normalized and 14% resulted in early pregnancy loss. Their follow-up management after the two-week follow-up was not detailed in their report. They did state that, of the 13 angular pregnancies that persisted, 11 migrated to a more central location [6].

Though most of these pregnancies result in a live delivery, these pregnancies can lead to uterine rupture, a rare and life-threatening pregnancy complication. In our literature review, we found wide variations of 0%, 12.6%, 13.6%, and 28% of angular pregnancies resulting in uterine rupture [3,5,6,10]. The largest study included 200 cases of angular pregnancies, but the study was done retrospectively, leading to possible selection bias [5].

Risk factors that predispose angular pregnancy patients to uterine rupture are a previous ipsilateral salpingectomy, vaginal bleeding, and gestational age of greater than seven weeks [5]. Low hemoglobin and blood transfusions were the most strongly correlated with uterine rupture in angular pregnancy patients [5]. Early fetal loss is a common sequela of angular pregnancies, with a statistically significant odds ratio of 4.0 when compared to pregnancies without angular pregnancy [12].

One study proposed a classification system of angular pregnancies of type I and type II. Type I angular pregnancies are located at the mediolateral junction of the uterine cavity and surrounded by two circular decidual bands with a partial myometrial border [5]. Type I angular pregnancies are generally safe and can be continued and monitored. Type II angular pregnancies expand laterally and carry a significantly higher risk of uterine rupture and maternal hemorrhage [5]. This classification system was designed as a tool for clinicians to utilize when considering observational management but has not been widely accepted.

## Conclusions

Identifying an angular pregnancy is essential to guide management and counsel patients. Angular pregnancies can result in a viable pregnancy, early fetal loss, or uterine rupture and must be differentiated from myometrial or cornual pregnancies. This wide range of outcomes makes accurate diagnosis essential.
